# Laparoscopic cholecystectomy for giant gallbladder: A case report

**DOI:** 10.1097/MD.0000000000035429

**Published:** 2023-10-06

**Authors:** Yi Gao, Diancai He, Wenqi Feng, Jing Yue, Zhiyuan Jian

**Affiliations:** a General Surgery I Department, Affiliated Hospital of Guilin Medical University, Guilin, Guangxi Province, China.

**Keywords:** giant gallbladder, laparoscopic cholecystectomy, magnetic resonance cholangiopancreatography

## Abstract

**Rationale:**

An overdistended gallbladder is usually observed in cases of distal bile obstruction due to malignancy. The gallbladder may also become enlarged and distended during cystic duct or gallbladder neck obstruction due to gallstones. However, a grossly distended gallbladder ( > 14 cm in length) without any pathology is rare. We present the case of a 46-year-old female patient who suffered from acute right lower quadrant pain for 4 days. Initially, a liver cyst and a choledochal cyst were diagnosed by the local hospital. Then, the diagnosis of giant gallbladder (measuring approximately 20.0 cm × 7.0 cm and containing more than 30 gallbladder stones) was made by magnetic resonance cholangiopancreatography at our hospital. Finally, we successfully performed a laparoscopic cholecystectomy and the patient had an uneventful recovery.

**Patient concerns:**

A 46-year-old female patient presented with acute right lower quadrant pain lasting 4 days. At first, the abdominal pain was severe and paroxysmal, and then it subsided spontaneously. Computed tomography of the abdomen at another hospital revealed a hepatic cyst and a choledochal cyst. Come to our hospital for surgical treatment.

**Diagnoses:**

giant gallbladder with gallstones.

**Interventions:**

Laparoscopic cholecystectomy was successfully performed in this patient after decompressing the gallbladder.

**Outcomes:**

On the third postoperative day, the patient recovered well, and the abdominal pain resolved following the operation. At the 3-year postoperative follow-up, the patient was symptom-free, with no obvious abnormalities seen in liver function and hepatobiliary color Doppler ultrasound.

**Lessons:**

The patient was successfully treated using laparoscopic cholecystectomy. This rare case may contribute to the development of mechanisms for treating giant gallbladders.

## 1. Introduction

The length of a normal gallbladder in adults is 7.5 to 10-cm.^[1]^ Giant gallbladder is usually defined as a gallbladder measuring > 14 cm in length.^[2]^ Giant gallbladder with no marked obstructive factors is very rare.^[3]^ Indeed, no more than 9 cases have been reported in the literature.^[4]^ The pathogenesis of the development of the giant gallbladder remains unknown. Usually, an increase in gallbladder volume can be due to physiological and pathological causes. The gallbladder itself can enlarge to a significant size without any functional abnormality or obstruction of the biliary tract. This physiological increase in gallbladder size usually does not require any treatment. The pathological causes of overdistended gallbladder are as follows: Mechanical obstruction of the cystic duct and common bile duct due to stone, tumor, dysmorphia, adhesion, ascaris, or external pressure; Bacterial infection of gallbladder; Gallbladder functional disorder of gallbladder due to biliary tract muscular or neural disorders; Diabetes mellitus; and; Other causes such as surgical trauma and pancreatic fluid reflux. Cholecystitis and cholelithiasis are the most common causes of gallbladder distension. We report a case of a giant gallbladder with gallstones in which its fundus reaches up to the level of the anterior superior iliac spine near the ileocecum. The patient was successfully treated using laparoscopic cholecystectomy (LC).

## 2. Case report

A 46-year-old female patient presented with acute right lower quadrant pain lasting 4 days. At first, the abdominal pain was severe and paroxysmal, and then it subsided spontaneously. She had no nausea, vomiting, fever, jaundice, or any other abdominal complaints. Computed tomography of the abdomen at another hospital revealed a hepatic cyst and a choledochal cyst. She was Gravida 3, Para 2 and underwent Cesarean section at another hospital in 2009. She denied alcoholism, smoking, drug use, and other illnesses.

At admission, the physical examination was unremarkable, except for the presence of a 10-cm healed surgical scar in the middle of the lower abdomen. A long tubular lump was palpable in the right side of the abdomen, with its lower border at the level of the anterior superior iliac spine. There was no significant tenderness or rebound tenderness.

**Figure 1.**
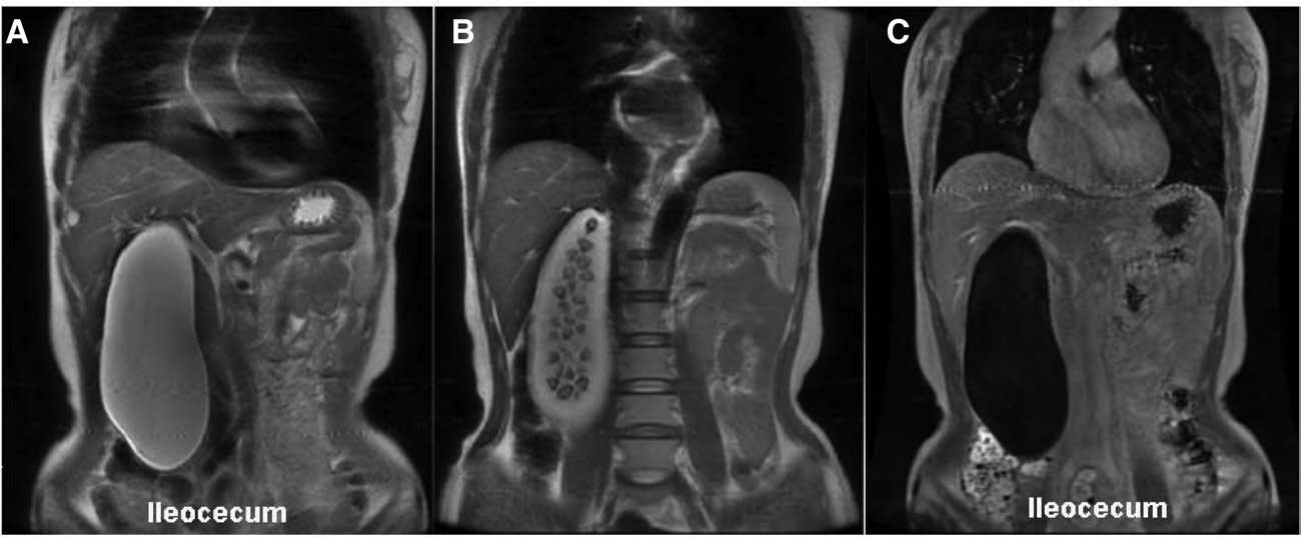
Magnetic resonance cholangiopancreatography (MRCP) showed a giant gallstone-containing gallbladder (20 × 7 cm) reaching up to the ileocecal region.

The routine blood test, coagulation function, blood biochemistry, tumor markers, and human chorionic gonadotropin levels were within normal limits (Table 1). An electrocardiogram revealed sinus rhythm with flattening of T-waves in V4, V5, and V6 leads. Abdominal magnetic resonance imaging showed that the gallbladder was markedly dilated, with a maximum transverse diameter of 7.0 cm and a longitudinal length of 20 cm. There were multiple stones within the gallbladder and a small cyst in the right anterior lobe of the liver (Fig. 1).

**Table 1:** Laboratory investigations upon admission.

Blood markers	Index	Normal range
Leucocytes (×10^9^/L)	3.89	3.5–9.5
Hemoglobin (g/L)	111	115–150
Platelets (×10^9^/L)	208	125–350
ALT (U/L)	8	0–40
AST (U/L)	14	0–35
GGT (U/L)	8	0–45
ALP (U/L)	64	35–100
TP (g/L)	70.1	65–85
ALB (g/L)	40.9	40–55
TBIL (μmol/L)	11.6	3.42–20.5
DBIL (µmol/L)	3.1	0–6.42
TBA (µmol/L)	4.3	0–10
GLU (mmol/L)	4.9	3.9–6.1
Urea (µmol/L)	3.37	1.7–7.5
Cr (µmol/L)	49	44–120
Ams (IU/L)	62	35–135
PA (mg/L)	165.2	170–420
PTA (%)	73.7	75–145
PT (sec)	13.2	9–13
APTT (sec)	31	25–37
Fbg (g/L)	2.29	2–4
HCG (IU/L)	2.3	0–10
CA125 (kU/L)	12.48	0–35
CA19-9 (kU/L)	15.1	0–37
AFP (μg/L)	4.05	0–15
CEA (μg/L)	1.34	0–10

ALT = alanine transaminase, AST = aspartate transaminase, ALP = alkaline phosphatase, GGT = g-glutamyl transferase, TBIL = total bilirubin, DBIL = direct bilirubin, TBA = total bile acids, GLU = glucose, ALB = albumin, AMs = amylase, PA = prealbumin, Cr = creatinine, BUN = blood urea nitrogen, PT = prothrombin time, APTT = activated partial thromboplastin time, Fbg = fibrinogen, HCG = human chorionic gonadotropin.

**Figure 2.**
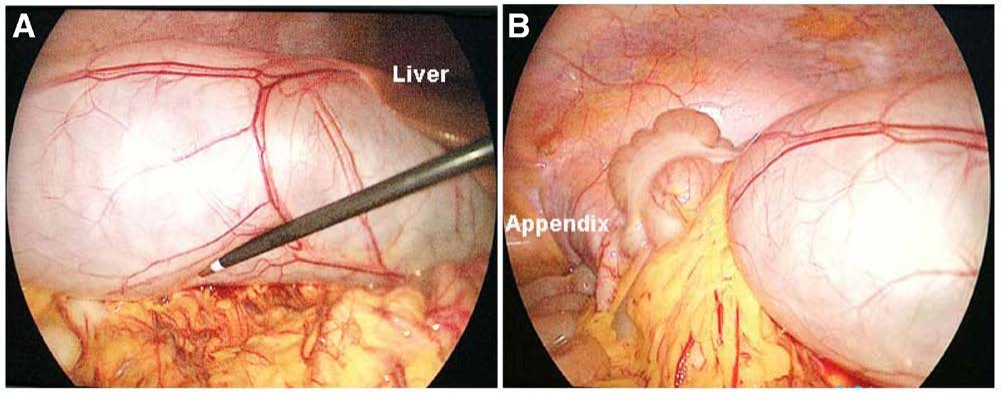
Intraoperative images showing the grossly distended gallbladder reaching up to the cecum and appendix.

The patient underwent LC under general anesthesia using a 10-mm supraumbilical port, a 10-mm subxiphoid port, and a 5-mm port at the level of the umbilicus in the right anterior axillary line. During the operation, the gallbladder was found to be grossly enlarged (20 cm × 7.0 cm). The lower boundary of the gallbladder reached the appendix (Fig. 2). The gallbladder wall was mildly edematous, thickened, and adhered to the greater omentum. The common bile duct was not dilated.

Since the gallbladder was too large to be operated on smoothly, it was decompressed, and 650 mL of thin yellow-green bile was aspirated. After decompression, adhesiolysis was performed, and the Calot triangle was dissected. The cystic artery and duct were carefully identified. The cystic artery was divided and coagulated near the gallbladder wall. The cystic duct was doubly clipped with Hem-o-lok clips about 0.5 cm from the common bile duct. The gallbladder was separated from the liver bed by a combination of anterograde and retrograde dissections. The operation took about 1 hour with minimal intraoperative blood loss.

**Figure 3.**
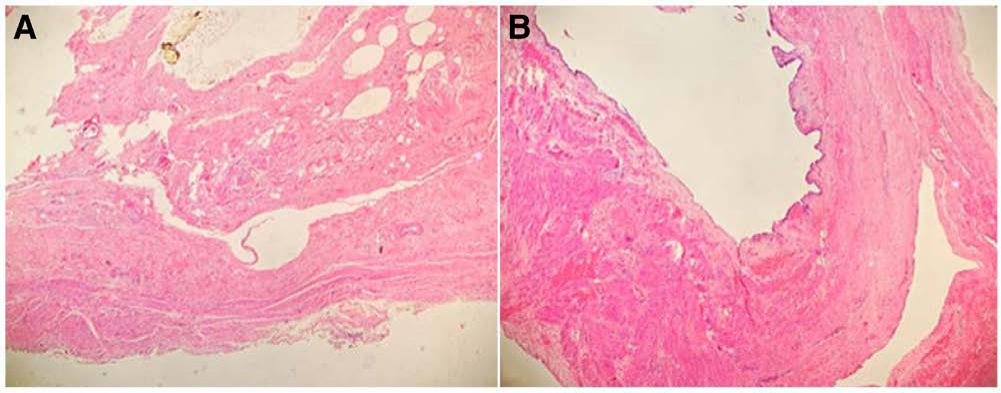
Pathological section with hematoxylin and eosin (HE) staining. (A) The gallbladder neck mucosa was ulcerated with fibrous hyperplasia of its wall, and there was no obvious inflammation. (B) The gallbladder fundus was normal, without obvious inflammation.

The postoperative recovery was uneventful with a hospital stay of 3 days. On postoperative pathological examination, the gallbladder wall was 0.2 to 0.3 cm thick and slightly thickened (up to 0.4 cm) at the gallbladder neck, and there were more than 30 calculi in the cavity (Fig. 3). Microscopic examination revealed chronic cholecystitis. At the 3-year postoperative follow-up, the patient was symptom-free, with no obvious abnormalities seen in liver function and hepatobiliary color Doppler ultrasound.

## 3. Discussion

Cholecystolithiasis, the most common biliary disorder with a prevalence of 11%,^[5]^ is more common in women. The gallbladder usually has a volume of about 60 mL and does not exceed 14 cm in length.^[1,6]^ Giant gallbladders are rare. Giant gallbladders are associated with various obstructions of the cystic duct of the bile duct, such as stones, parasites, or tumors.^[1,7,8]^ There are also sporadic reports of gallbladder neck gangliocytopenia leading to dilated gallbladder.^[6]^

In this patient, there was no cystic duct obstruction or any other underlying disease. Postoperative pathological examination showed chronic calculous cholecystitis similar to routine cases of cholelithiasis. The gallbladder contained thin yellow-green bile, which ruled out complete luminal obstruction due to stones, but there may have been intermittent and repeated obstruction of the gallbladder neck by the gallstones, resulting in compensatory expansion of the gallbladder. The congenital giant gallbladder should also be considered due to the absence of obvious pathology in this patient.^[9]^

Cholecystectomy, the treatment of choice for cholecystitis and gallstones, was first described by Langebuch in 1882. LC was first described by French surgeon Philippe Mouret in 1987. After more than 30 years of development, LC has become the gold standard for the treatment of symptomatic gallbladder disease. Indications for LC include recurrent biliary colic and the development of gallstone-related complications.

LC is recommended for asymptomatic gallstones under the following conditions: Many stones and stone diameter ≥ 2 to 3 cm; Gallbladder wall calcification or porcelain gallbladder; Gallbladder polyps (diameter ≥ 1 cm); Gallbladder wall thickness > 3 mm (accompanied by chronic cholecystitis).

Giant gallbladders are rare. This patient had a huge gallbladder complicated by multiple gallstones. LC was successfully performed in this patient after decompressing the gallbladder. The patient recovered well, and the abdominal pain resolved following the operation. This rare case may contribute to the development of mechanisms for treating giant gallbladders.

## Acknowledgements

We thank Tieyan Wang for technical assistance and Medjaden Inc. for the scientific editing of this manuscript.

## Author contributions

**Conceptualization:** Yi Gao.

**Supervision:** Wenqi Feng, Jing Yue.

**Validation:** Diancai He, Wenqi Feng.

**Visualization:** Jing Yue.

**Writing – original draft:** Yi Gao, Diancai He.

**Writing – review & editing:** Zhiyuan Jian.
